# Supplementary material for: Major proliferation of transposable elements shaped the genome of the soybean rust pathogen Phakopsora pachyrhizi
Source: Nat Commun. 2023 Apr 1;14:1835. doi: 10.1038/s41467-023-37551-4 (PMC10067951; doi:10.1038/s41467-023-37551-4)
Supplement: Supplementary file 1 — Supplementary Information [file 41467_2023_37551_MOESM1_ESM.pdf]

Supplementary information

Major proliferation of transposable elements shaped the genome of the soybean rust pathogen *Phakopsora pachyrhizi*

Gupta *et al.*

Supplementary Figures

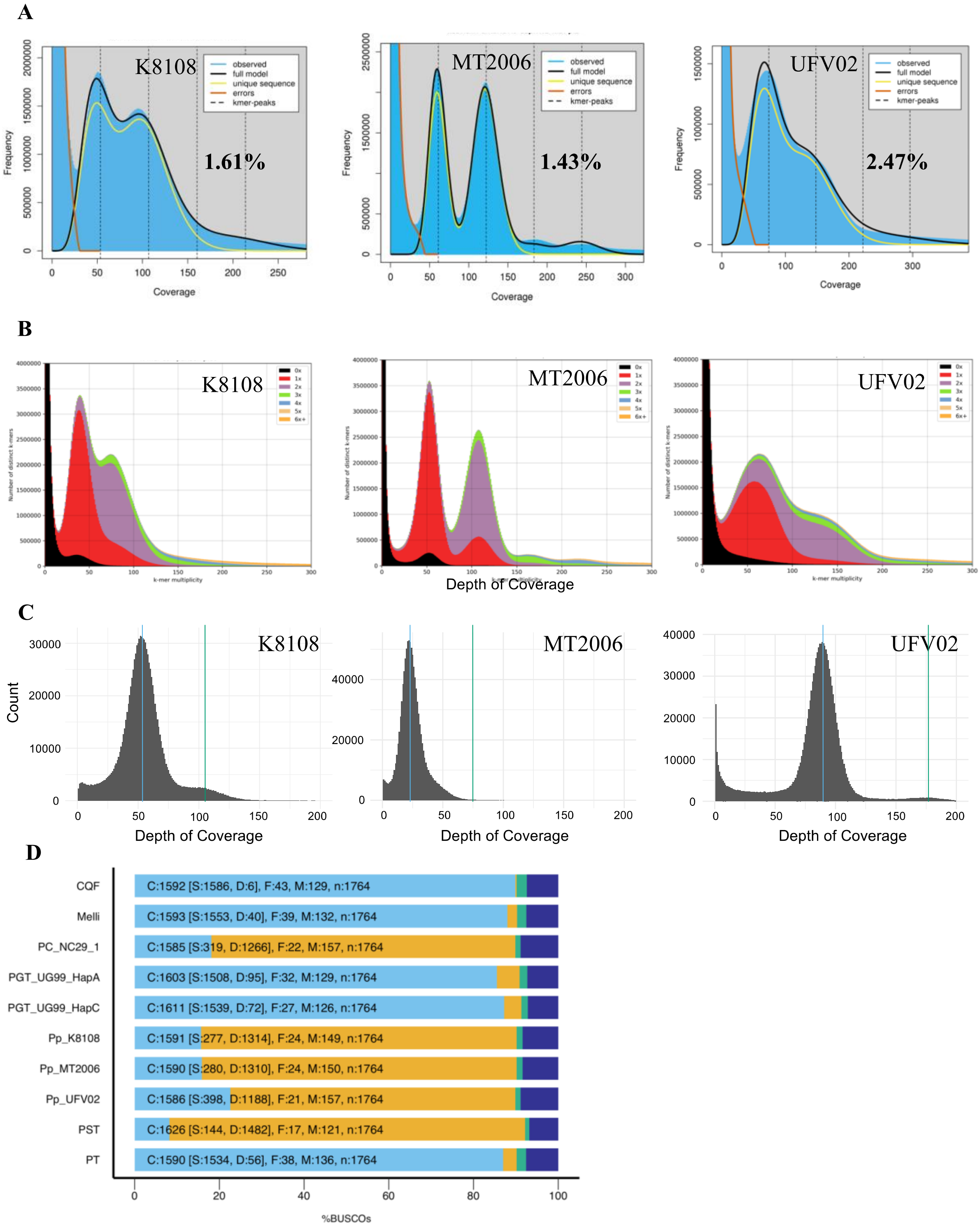

Supplementary Fig. 1. Comparison between the *P. pachyrhizi* genomes K8108, MT2006 and UFV02.

a K-mer frequency plots generated with WGS Illumina data of three isolates. The k-mer frequency was estimated using Jellyfish and GenomeScope2. The x-axis shows k-mer coverage and y-axis show the frequency. Two peaks in K-mer frequency profile shows a high level of heterozygosity in *P. pachyrhizi*. The level of heterozygosity (shown in the bold letters) varied between 1.43 to 2.47%. **b** K-mer spectra plot comparing k-mer content of Illumina read to k-mer content of the respective genomes, where different colors represent the number of times k-mers from the reads found in the genome assembly. Black: indicates k-mer content present in the raw reads but missing the genome assembly. Red: K-mers present in the reads and once in the assembly. Purple: K-mers present in the reads and twice in the genome assembly. Other colors indicate k-mers present in the genome more than twice. **c** Raw read-depth histograms of K8108, MT2006 and UFV02, the main peak (blue) showing non-collapsed reads at 1x haploid coverage and the secondary peak (green) at 2x haploid coverage. **d** BUSCO analysis of three *P. pachyrhizi* genomes and comparison with the genomes of other published rust fungi. The basidiomycota database (n=1764) was used for the BUSCO analysis.

Abbreviations: *Cronartium quercuum* f. sp. *fusiforme* G11 (CQF), *Melampsora lini* CH5 (Melli), *Puccinia coronata* f. sp. *avenae* 12NC29 (PC_NC29_1), *Puccinia graminis* f. sp. *tritici* UG99 haplotype A (PGT_UG99_HapA), *Puccinia graminis* f. sp. *tritici* UG99 haplotype C (PGT_UG99_HapC), *P. pachyrhizi* K8108 (Pp_K8108), *P. pachyrhizi* MT2006 (Pp_MT2006), *P. pachyrhizi* UFV02 (Pp_UFV02), *Puccinia striiformis* f. sp. *tritici* PST-130 (PST), *Puccinia triticina* Pt76 (PT).

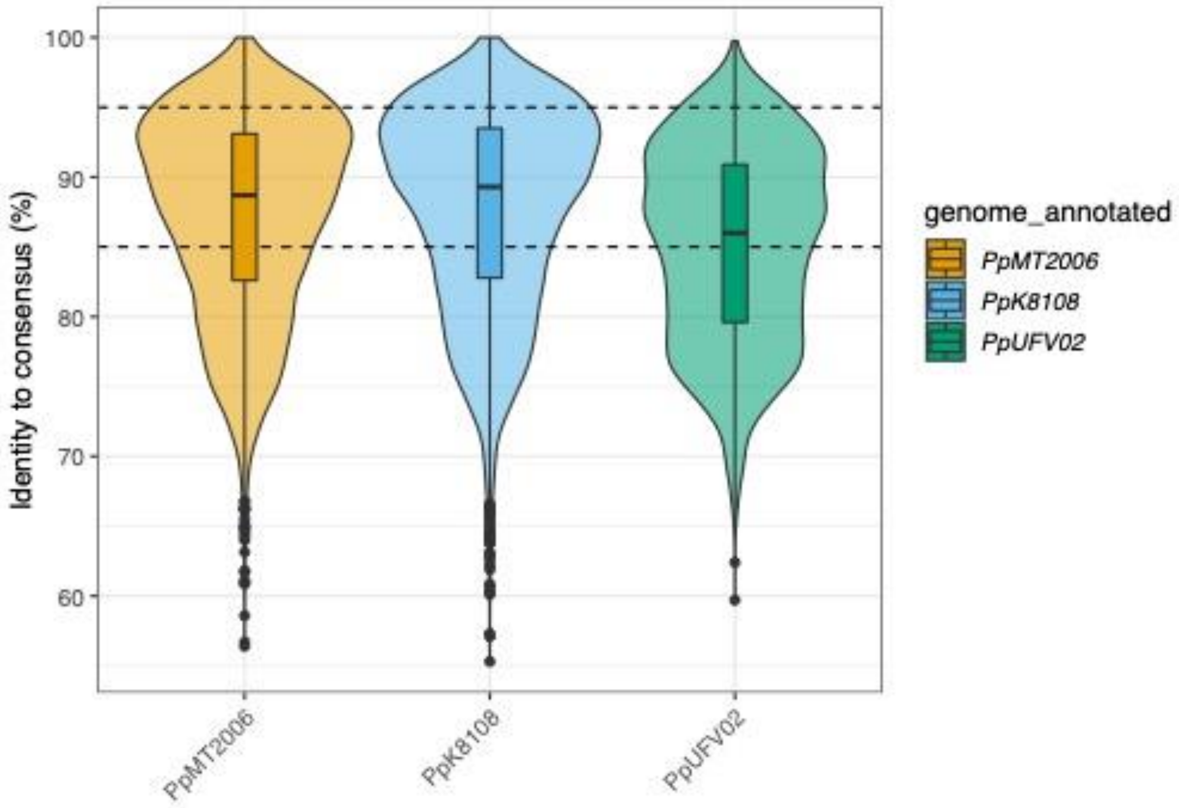

Supplementary Fig. 2. TE consensus identity in the *P. pachyrhizi* genomes K8108, MT2006 and UFV02. Based on the sequence identity, TEs were categorized as (1) conserved TEs (copies with more than 95% identity), (2) intermediate TEs (copies with 85 to 95% identity) and (3) divergent TEs (copies with less than 85% identity). The dotted line represents the cutoff for the sequence identity. Violin plots indicate: vertical line represents distribution at Q1-1.5 × IQR and Q3+ 1.5 × IQR, dots represent independent data points, first quartile (lower bar), median (thick line), third quartile (upper bar), and the shape indicates the frequency. Source data are provided as a Source Data file.

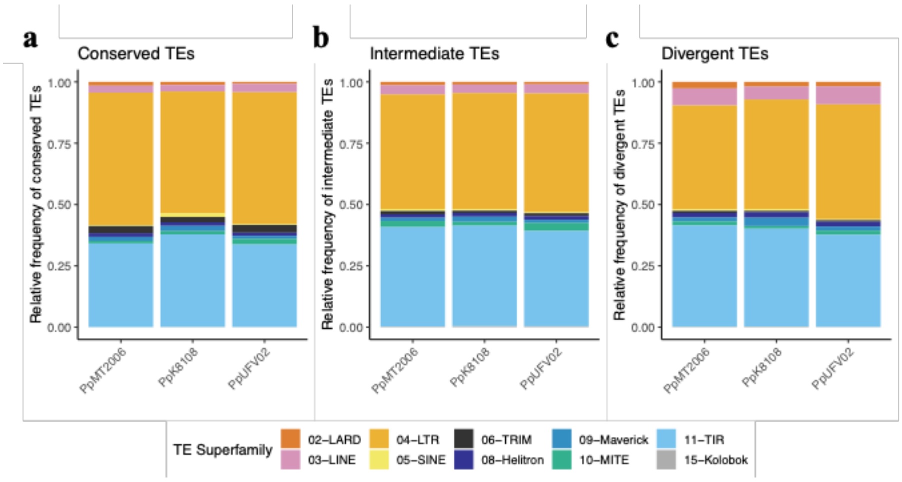

Supplementary Fig. 3. Relative frequency of TE superfamilies in different categories. **a** Conserved TEs, **b** Intermediate TEs, and **c** Divergent TEs in the *P. pachyrhizi* genomes K8108, MT2006 and UFV02. Source data are provided as a Source Data file.

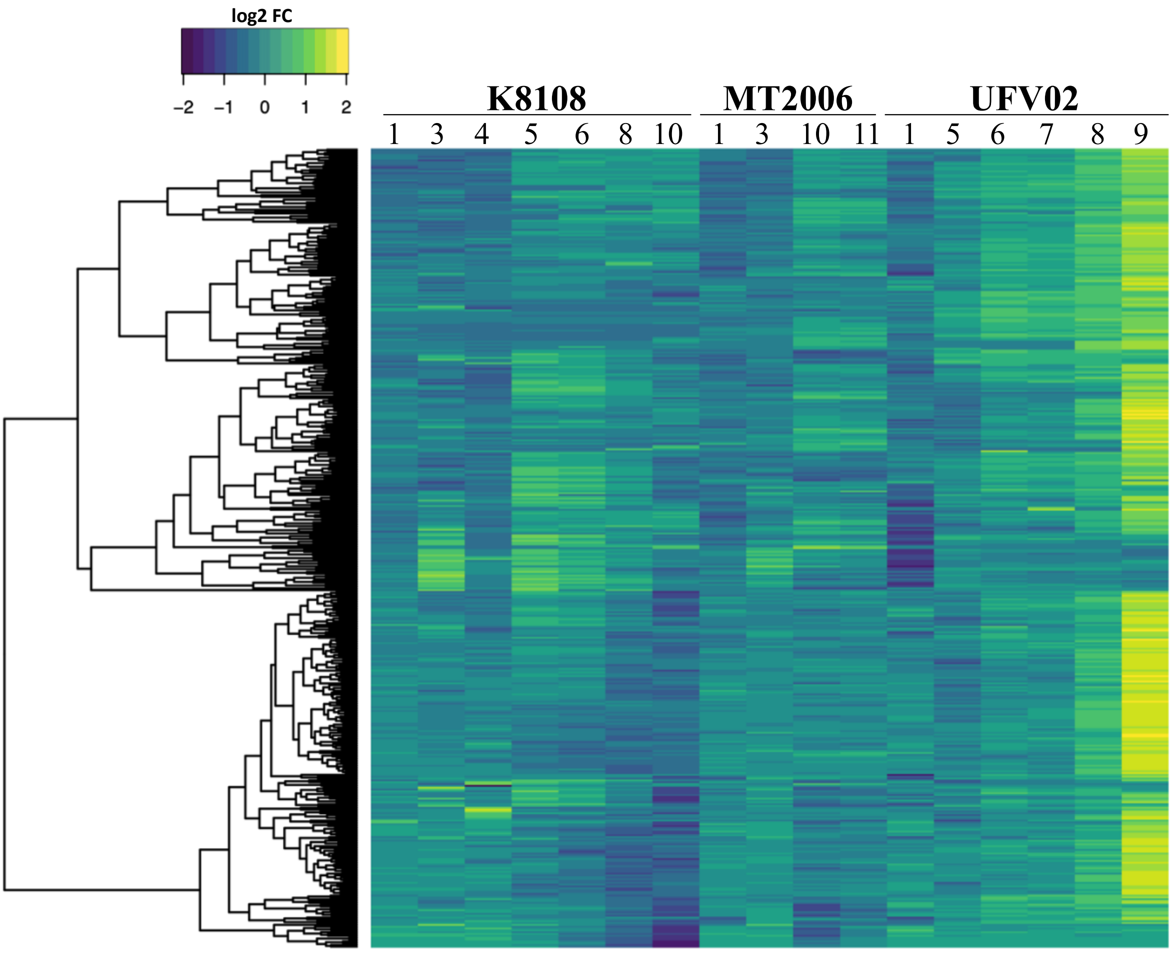

Supplementary Fig. 4. Heatmap of differentially expressed genes encoding predicted secreted proteins from the *P. pachyrhizi* genomes K8108, MT2006 and UFV02.
DEGs were hierarchical clustered by treatment, applying hclust method using R package (*150*). Differentially expressed genes in the different conditions (1) Spore; (3) appressorium *in vitro*; (4) appressorium *in planta*; (5) 10-12 HPI; (6) 24 HPI; (7) 36 HPI; (8) 72 HPI; (9) 168 HPI; (10) 192-196 HPI; (11) 288 HPI relative to the germinated spores. The scale bar shows log2 fold-change.

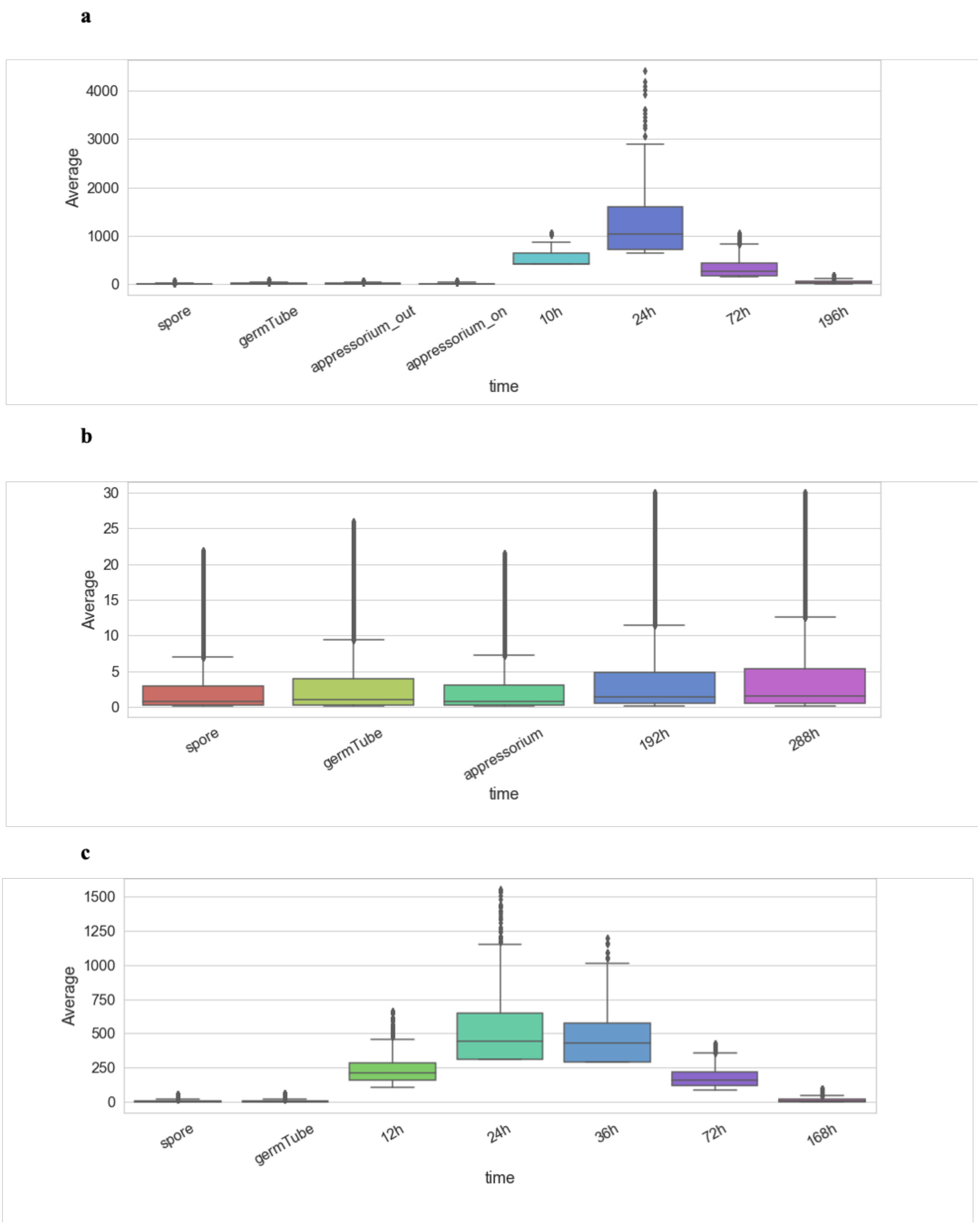

Supplementary Fig. 5. Expression profile of TEs on different condition in the *P. pachyrhizi* transcriptomes.

Average of CPM (copies per million) of TEs. **a** K8108, **b** MT2006, and **c** UFV02. Box plots indicate: vertical line represents distribution, dots represent outliers, first quartile (lower bar), median (thick line), third quartile (upper bar), the box indicates the interquartile range (IQR), lower whisker is first quartile - 1.5 × IQR and upper whisker is third quartile + 1.5 × IQR. (n= three independent biological replicates). Source data are provided as a Source Data file.

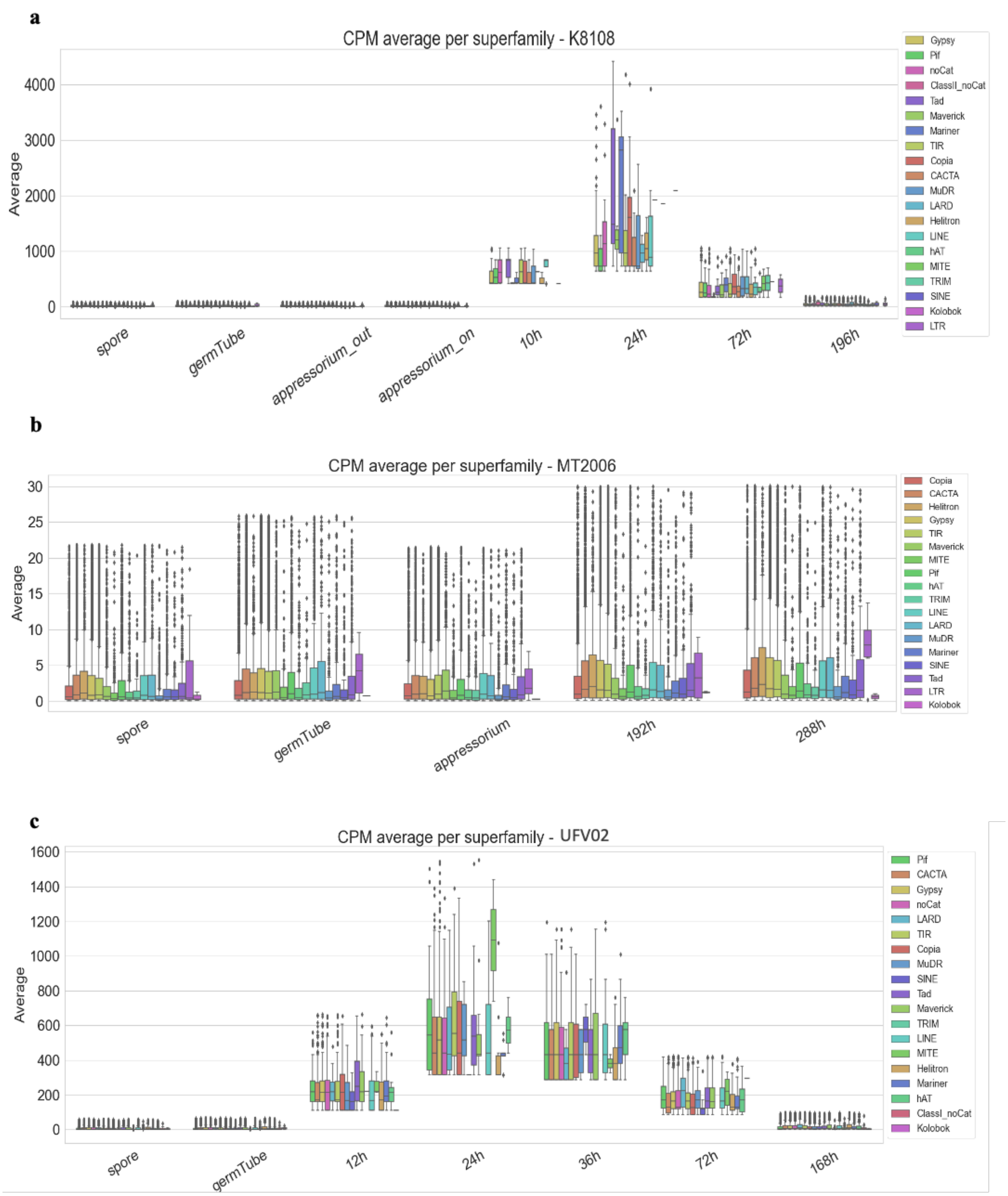

Supplementary Fig. 6. Expression profile of TEs in superfamilies different conditions (mentioned in Fig. 2) in the *P. pachyrhizi* transcriptomes.

Average of CPM (copies per million) of TEs. **a** K8108, **b** MT2006, and **c** UFV02. Box plots indicate: dots represent outliers, first quartile (lower bar), median (thick line), third quartile (upper bar), the box indicates the interquartile range (IQR), lower whisker is first quartile - 1.5 × IQR and upper whisker is third quartile + 1.5 × IQR. (n= three independent biological replicates). Source data are provided as a Source Data file.

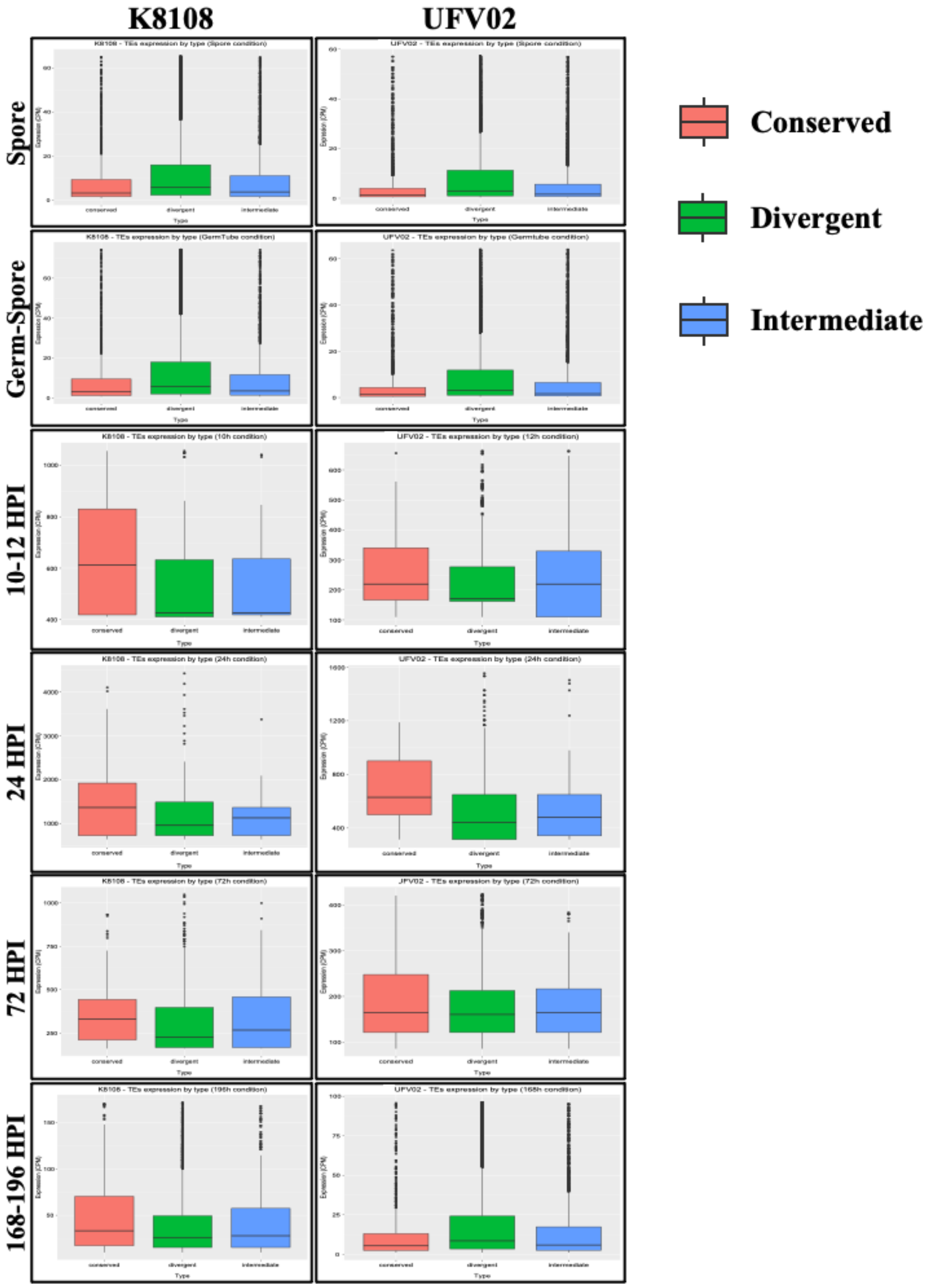

Supplementary Fig. 7. Expression profile of TEs based on conserved, divergent, and intermediate categories in the *P. pachyrhizi* genomes K8108 and UFV02.

Average of CPM (copies per million) of TEs. Box plots indicate: Box plots indicate: dots represent outliers, first quartile (lower bar), median (thick line), third quartile (upper bar), the box indicates the interquartile range (IQR), lower whisker is first quartile - 1.5 × IQR and upper whisker is third quartile + 1.5 × IOR. (n= three independent biological replicates). Source data are provided as a Source Data file.

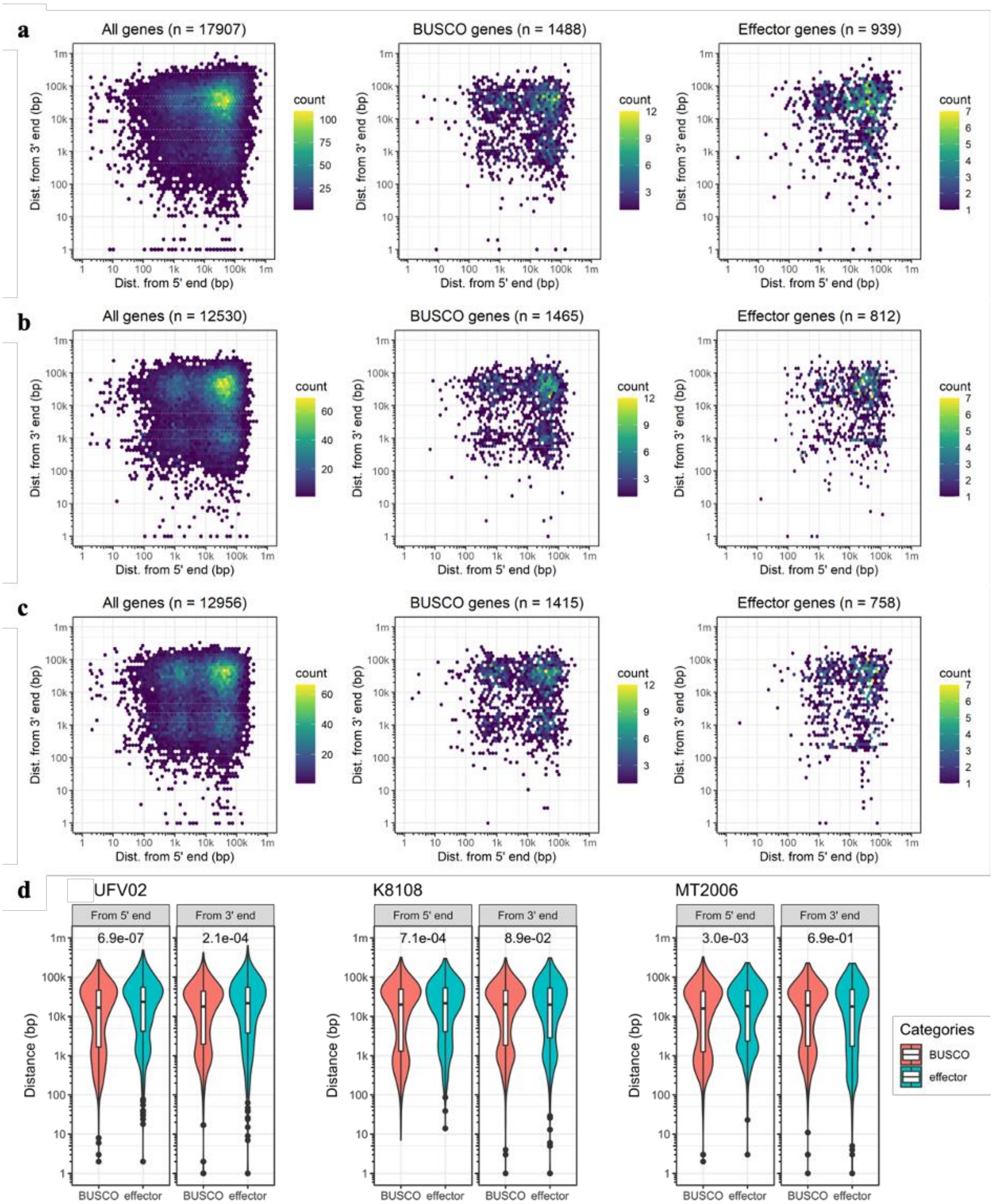

Supplementary Fig. 8. Distribution of effector genes in comparison with gene catalogues and BUSCO genes in the *P. pachyrhizi* genomes K8108, MT2006 and UFV02.

a-c. Hexbin plots for 5'(x-axis) and 3'(y-axis) intergenic distances. The left-most column represents profiles for all genes, the middle column for BUSCO genes, and the right-most column for effector genes. The number of genes included in the analysis (genes with both flanks within the same contig) is indicated in the parenthesis. **a** UFV02; **b** K8108; **c** MT2006. **d** Violin plots for 5' and 3' intergenic distances of BUSCO and effector genes. *P* values from two-sided Wilcox test are indicated in the plots. For each category, the number of genes included in the analysis are represented in a-c. The basidiomycota_odb10 dataset was used for the BUSCO analysis. Violin plots indicate: vertical line represents distribution at Q1-1.5 × IQR and Q3+

1.5 × IQR, dots represent independent data points, first quartile (lower bar), median (thick line), third quartile (upper bar), and the shape indicates the frequency. (n = one independent biological sample). Source data are provided as a Source Data file.

a

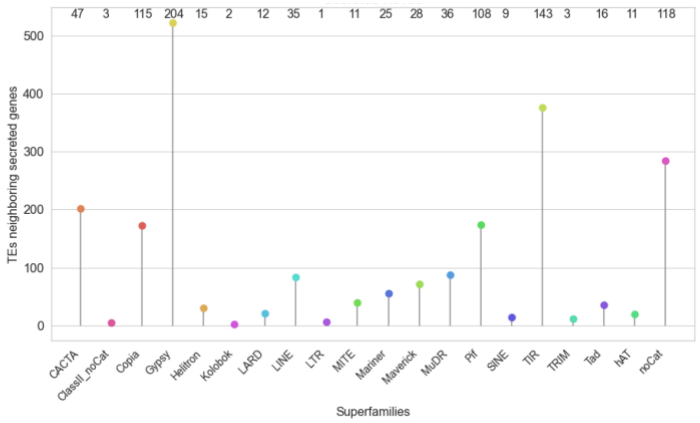

b

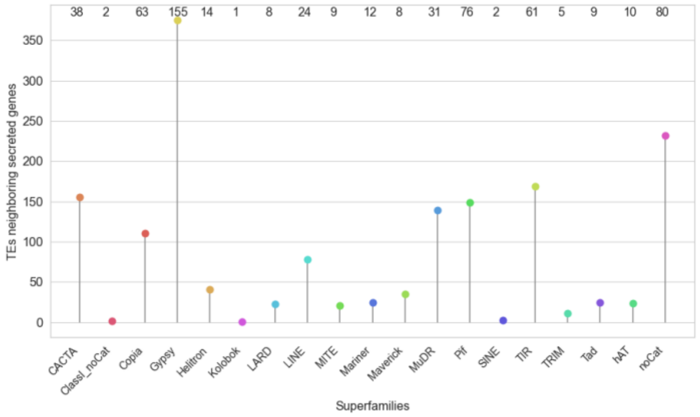

c

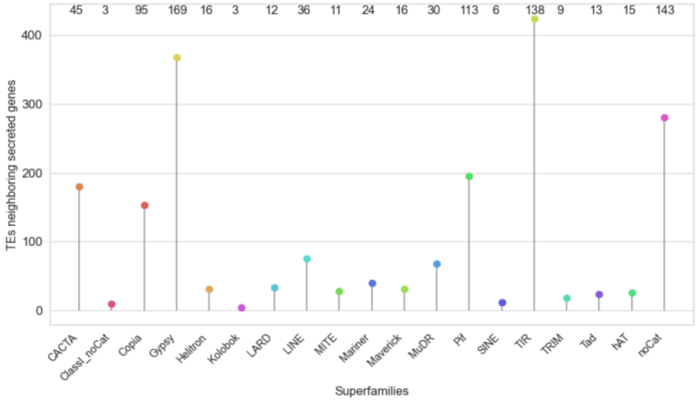

Supplementary Fig. 9. Association of secreted genes to the neighboring TE families in the *P. pachyrhizi* genomes. a K8108, b UFV02, and c MT2006.

The number of secreted genes correspond to the TE families shown at the top of the plot. Source data are provided as a Source Data file.

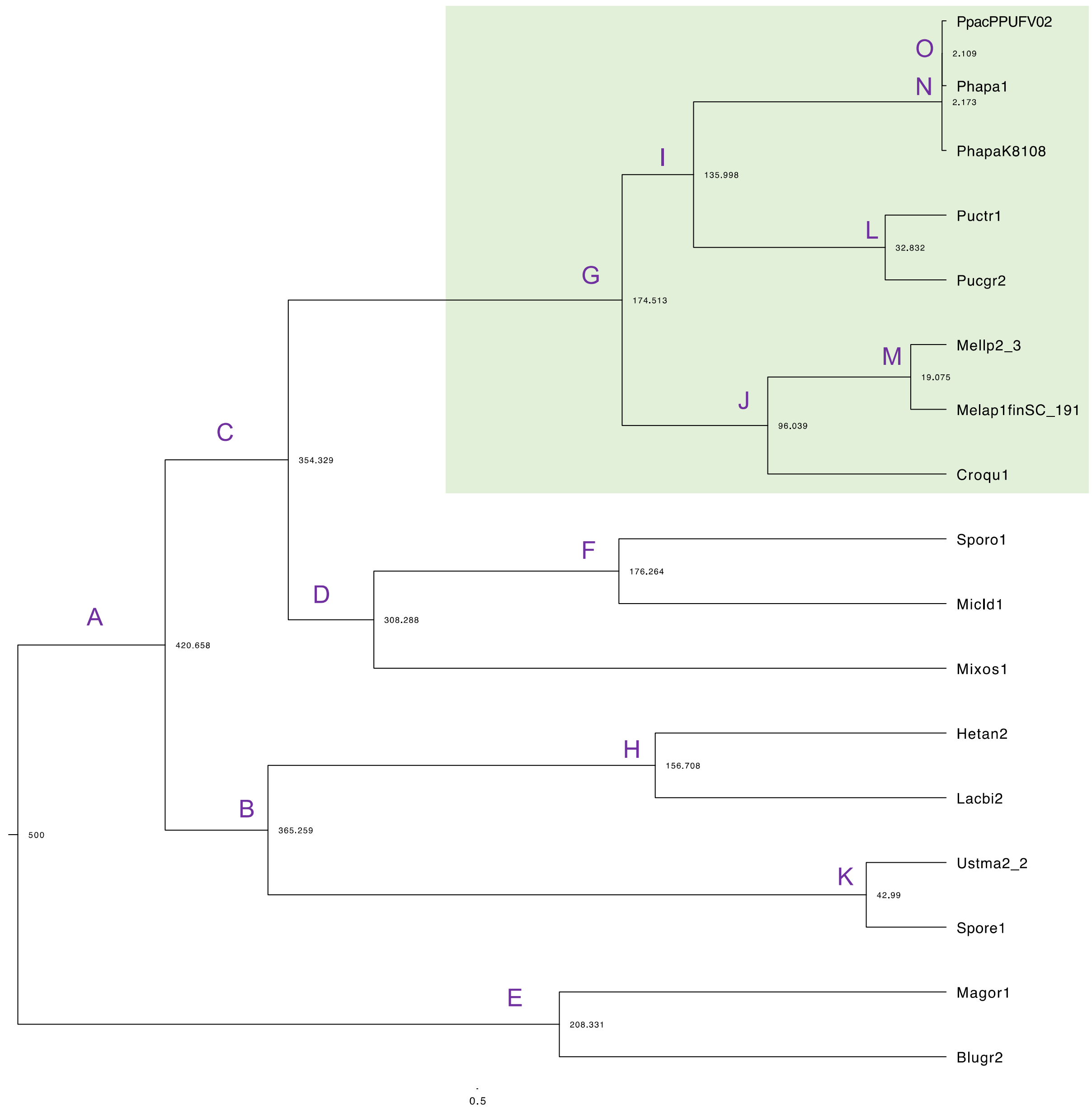

Supplementary Fig. 10. Phylogenetic relationships and estimated divergence time of selected fungal species.

The phylogenetic tree was generated after alignment of 408 conserved orthologous markers identified from at least 13 genomes using PHYling (Supplementary Data 24a). The sequences were aligned and concatenated into a super-alignment with 408 partitions. Phylogenetic tree was built with RAxML-NG (v0.9.0) using a partitioned analysis and 200 bootstraps replicates. The divergence times are indicated at the nodes in millions of years (My). The scale bar, 0.5 My is shown at the bottom of the phylogenetic tree.

Abbreviations: *P. pachyrhizi* MG2006 v1.0 (Phapa1), *P. pachyrhizi* K8108 v2.0 (PhapaK8108), *P. pachyrhizi* UFV02 v2.1 (PpacPPUFV02), *Cronartium quercuum* f. sp. *fusiforme* G11 (Croqu1), *Melampsora laricis-populina* v2.0 (Mellp2_3), *M. allii-populina* 12AY07 v1.0 (Melap1finSC_191), *P. graminis* f. sp. *tritici* v2.0 (Pucgr2), *P. triticina* 1-1 BBBD Race 1 (Puctr1), *Sporobolomyces roseus* v1 (Sporo1), *Mixia osmundae* (Mixos1), *Microbotryum lychnidis-dioicae* p1A1 Lamole (Micld1), *Ustilago maydis* 521 v2.0 (Ustma2_2), *Sporisorium reilianum* SRZ2 (Spore1), *Laccaria bicolor* v2 (Lacbi2), *Heterobasidion annosum* TC 32-1 (Hetan2), *Blumeria graminis* f. sp. *hordei* DH14 (Blugr2), *Magnaporthe oryzae* 70-15 v3.0 (Magor1).

a

b

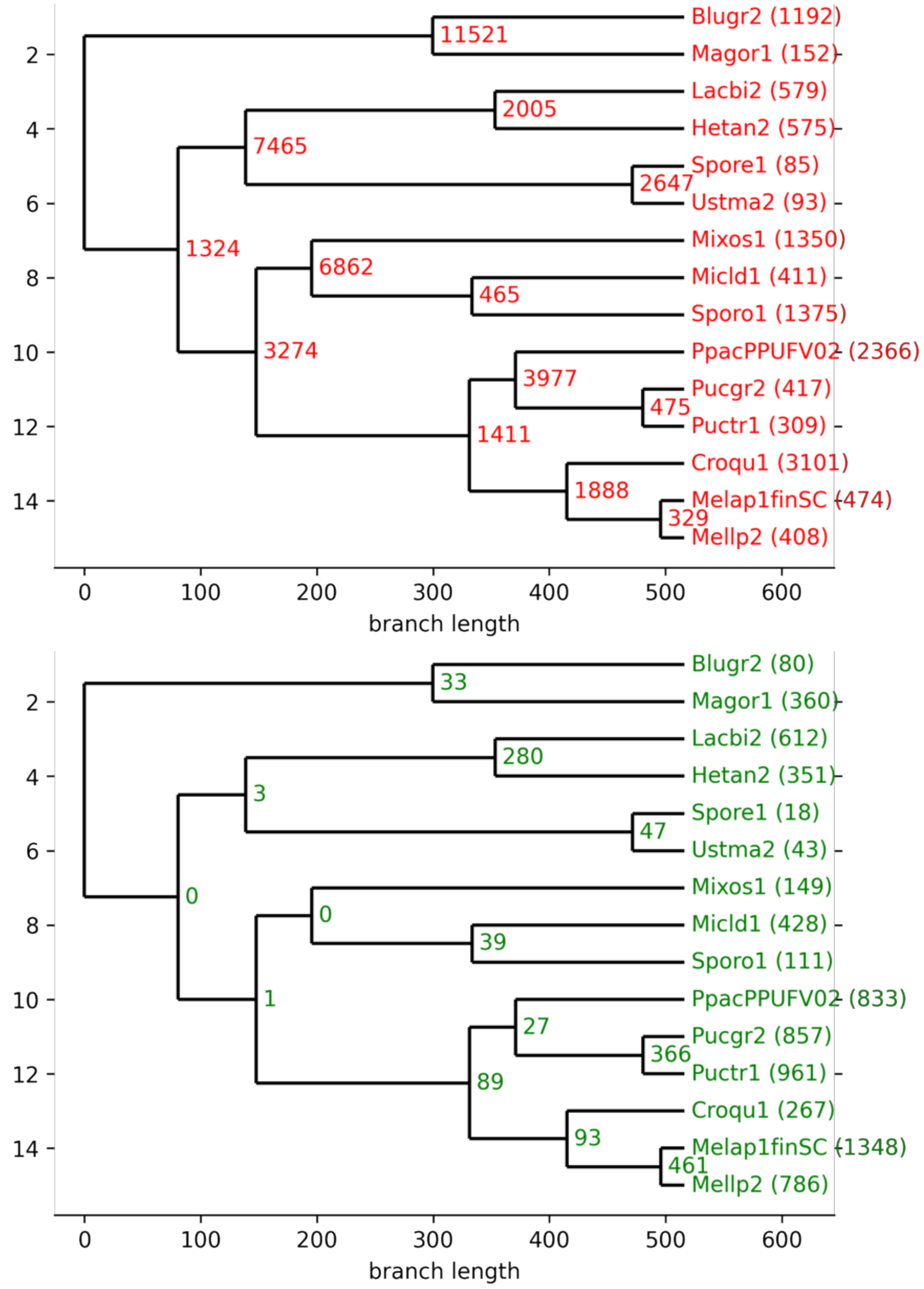

Blugr2 (1192)
Magor1 (152)
Lacbi2 (579)
Hetan2 (575)
Spore1 (85)
Ustma2 (93)
Mixos1 (1350)
Micld1 (411)
Sporo1 (1375)
PpacPPUFV02 (2366)
Pucgr2 (417)
Puctr1 (309)
Croqu1 (3101)
Melap1finSC (474)
Mellp2 (408)
11521
2005
7465
2647
1324
6862
465
3274
3977
475
1411
1888
329
branch length
Blugr2 (80)
Magor1 (360)
Lacbi2 (612)
Hetan2 (351)
Spore1 (18)
Ustma2 (43)
Mixos1 (149)
Micld1 (428)
Sporo1 (111)
PpacPPUFV02 (833)
Pucgr2 (857)
Puctr1 (961)
Croqu1 (267)
Melap1finSC (1348)
Mellp2 (786)
33
280
3
47
0
0
39
1
27
366
89
93
461
branch length

Supplementary Fig. 11. Gene family analysis shows contraction (a) and expansion (b) in 15 different fungal pathogens.

The branch length represents differentiation time. Number of expanded and contracted gene families are shown after the species name. The numbers on the nodes correspond to the ancestral protein families.

Abbreviations: *P. pachyrhizi* UFV02 v2.1 (PpacPPUFV02), *Cronartium quercuum* f. sp. *fusiforme* G11 (Croqu1), *Melampsora laricis-populina* v2.0 (Mellp2_3), *M. allii-populina* 12AY07 v1.0 (Melap1finSC_191), *P. graminis* f. sp. *tritici* v2.0 (Pucgr2), *P. triticina* 1-1 BBBD Race 1 (Puctr1), *Sporobolomyces roseus* v1 (Sporo1), *Mixia osmundae* (Mixos1), *Microbotryum lychnidis-dioicae* p1A1 Lamole (Micld1), *Ustilago maydis* 521 v2.0 (Ustma2_2), *Sporisorium reilianum* SRZ2 (Spore1), *Laccaria bicolor* v2 (Lacbi2), *Heterobasidion annosum* TC 32-1 (Hetan2), *Blumeria graminis* f. sp. *hordei* DH14 (Blugr2), *Magnaporthe oryzae* 70-15 v3.0 (Magor1)**.**

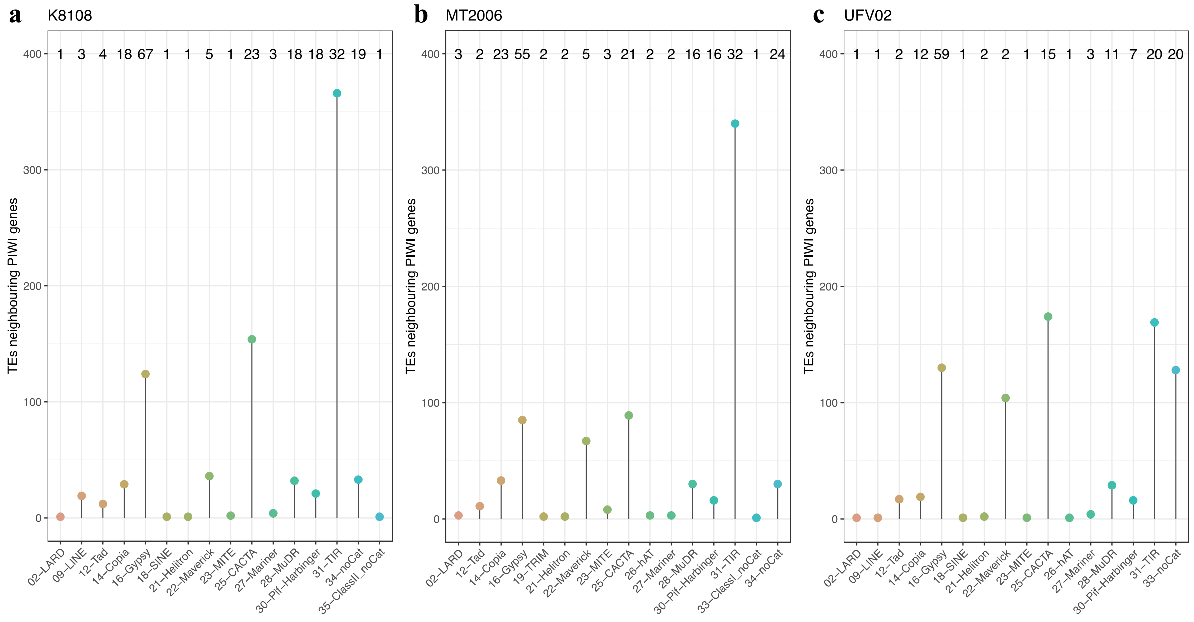

Supplementary Fig. 12. Association of Piwi genes to the neighboring TE families in the *P. pachyrhizi* genomes. a K8108, b MT2006, and c UFV02.

The number of Piwi genes correspond to the TE families shown at the top of the plot. Source data are provided as a Source Data file.

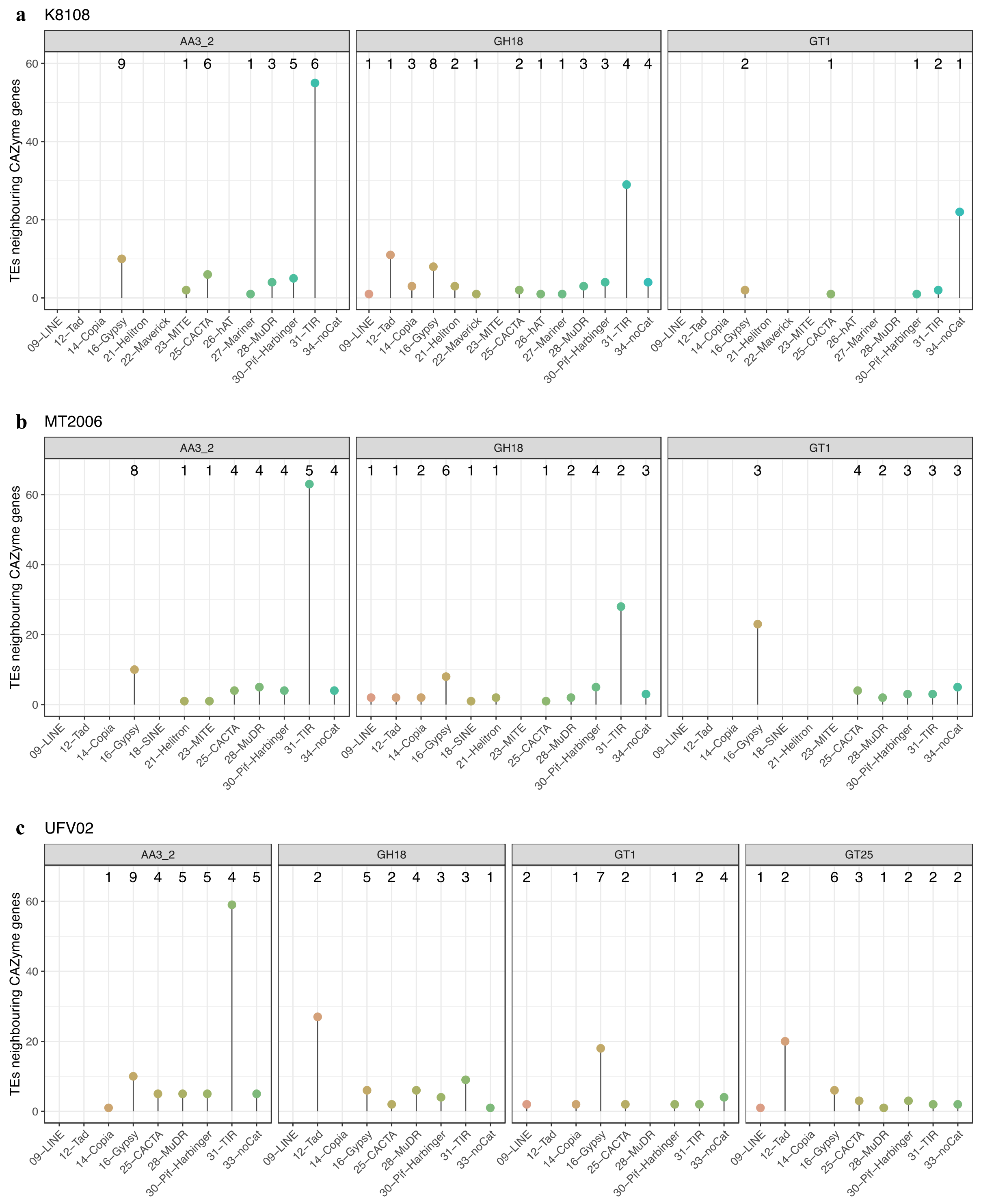

Supplementary Fig. 13. Association of CAZyme related genes to the neighboring TE families in the *P. pachyrhizi* genomes. a K8108, b MT2006, and c UFV02.
The number of CAZyme correspond to the TE families shown at the top of the plot. Source data are provided as a Source Data file.

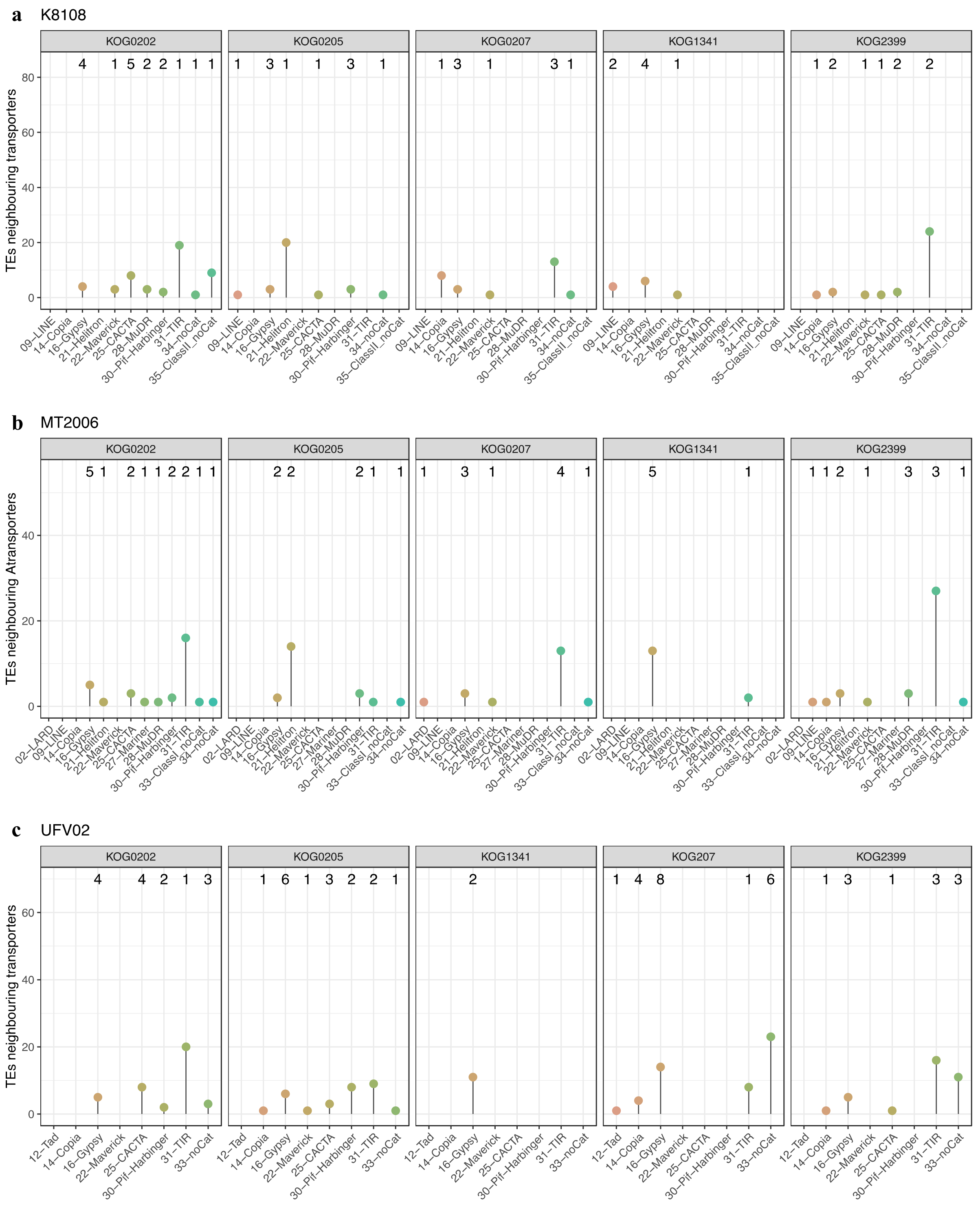

Supplementary Fig. 14. Association of transporter related genes to the neighboring TE families in the *P. pachyrhizi* genomes. a K8108, b MT2006, and c UFV02.
The number of transporter related genes correspond to the TE families shown at the top of the plot. Source data are provided as a Source Data file.

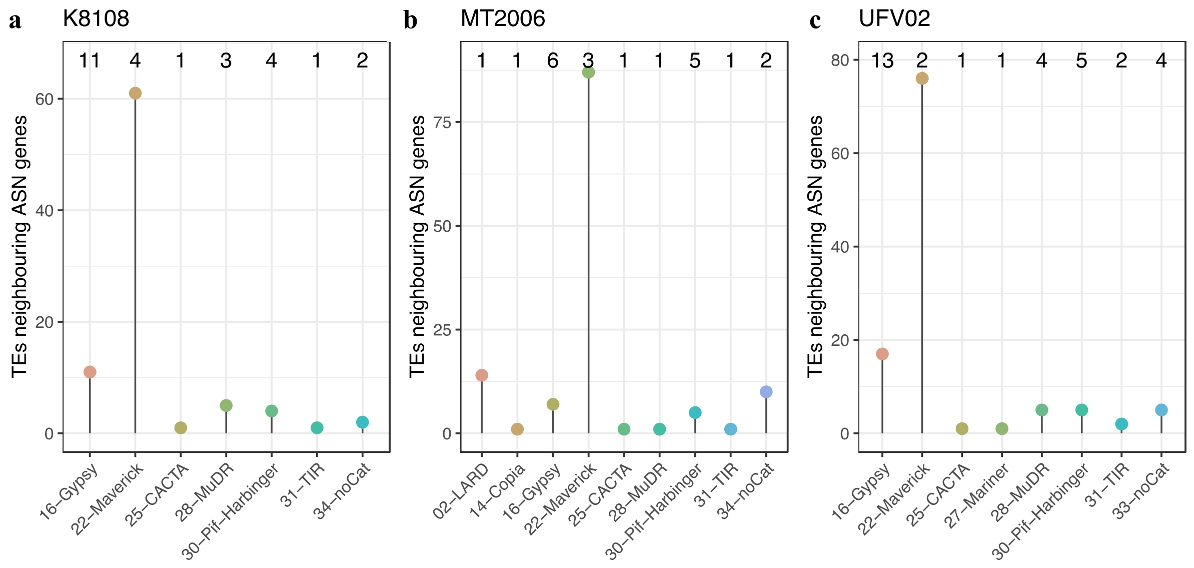

Supplementary Fig. 15. Association of Asparagine synthase (KOG0573) metabolism genes to the neighboring TE families in the *P. pachyrhizi* genomes. a K8108, b MT2006, and c UFV02.

The number of asparagine synthase genes correspond to the TE families shown at the top of the plot. Source data are provided as a Source Data file.

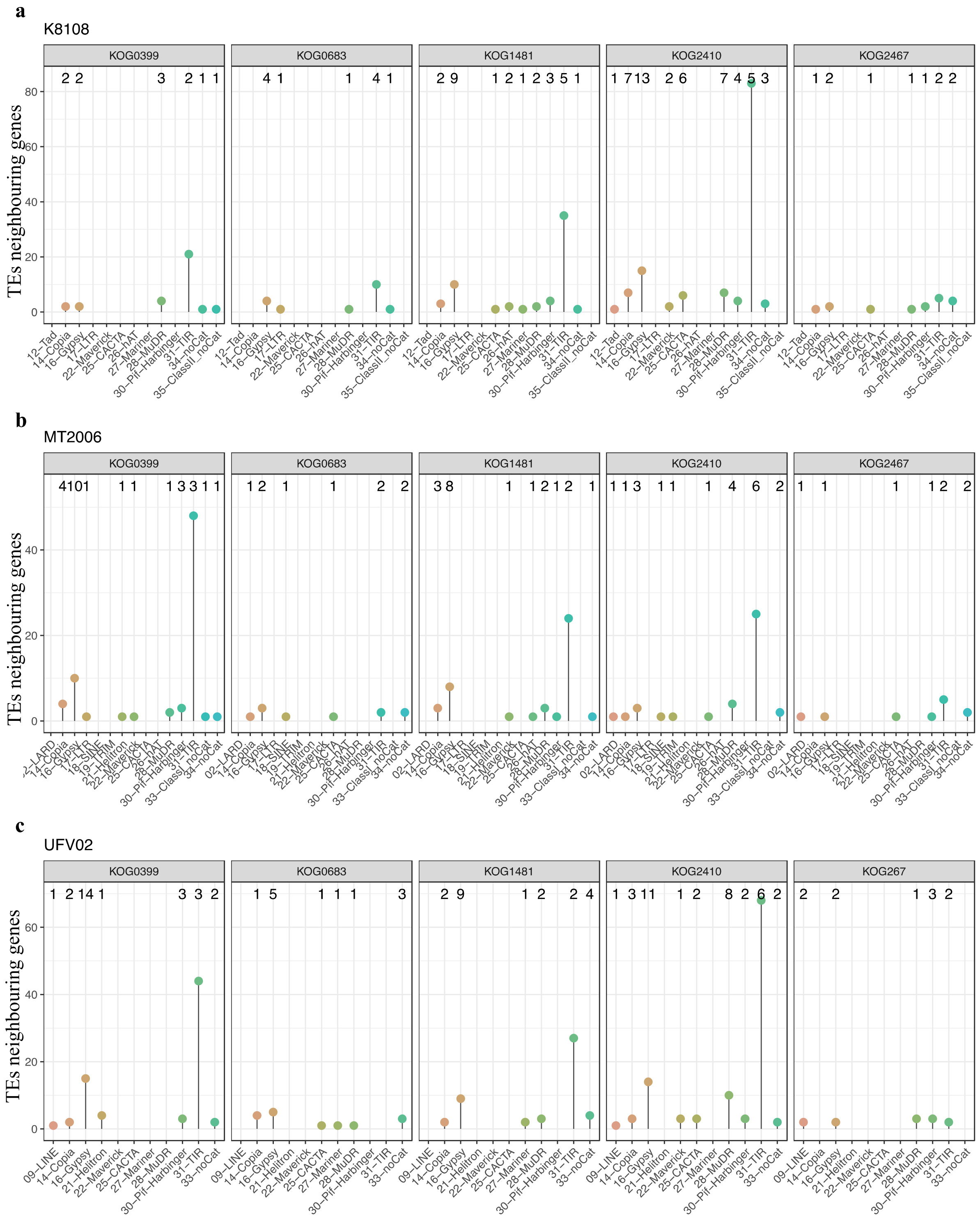

Supplementary Fig. 16. Association of amino acid metabolism genes to the neighboring TE families in the *P. pachyrhizi* genomes. a K8108, b MT2006, and c UFV02.
The number of amino acid metabolism genes correspond to the TE families shown at the top of the plot. Source data are provided as a Source Data file.

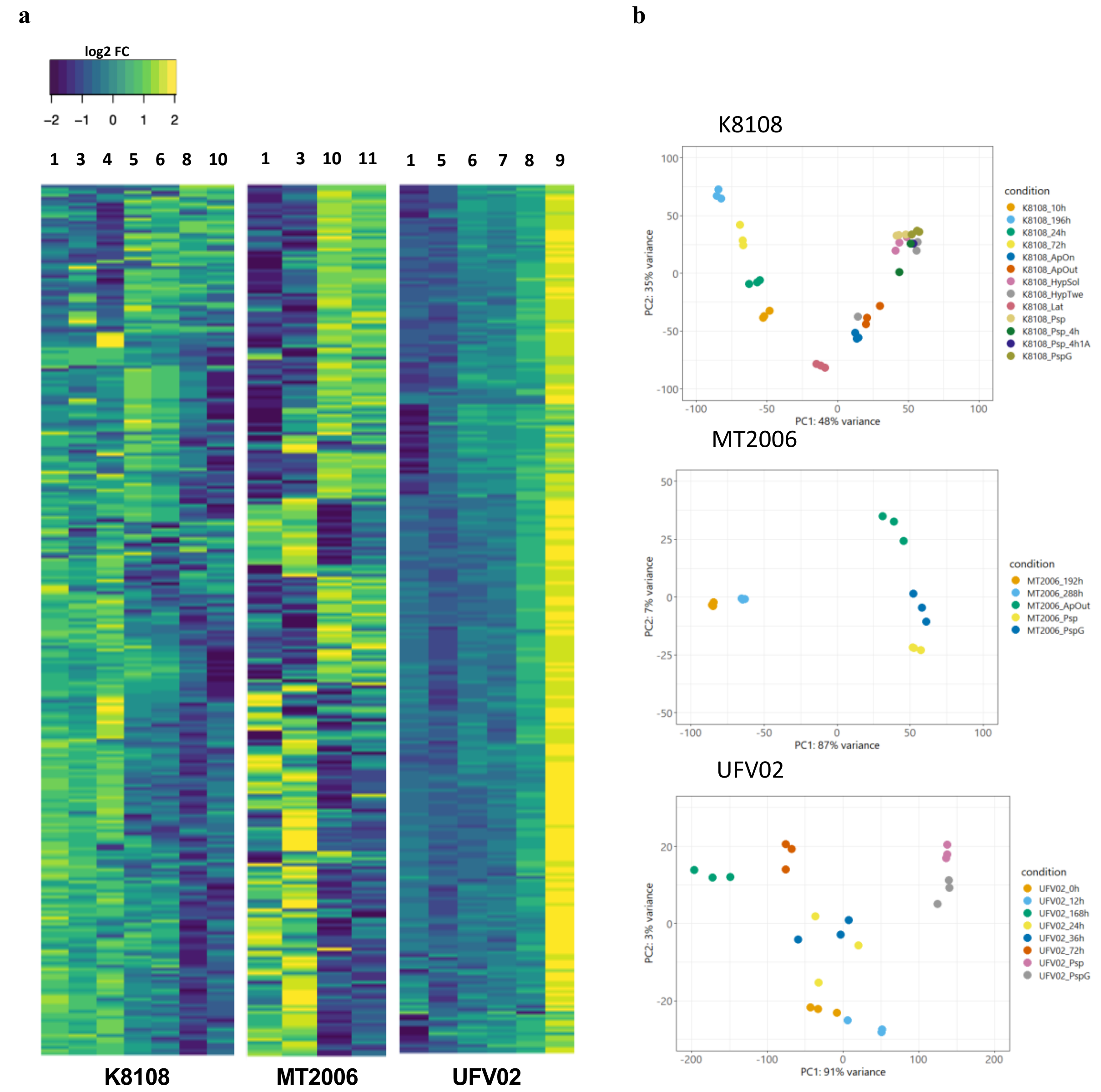

Supplementary Fig. 17. Heatmap of differentially expressed genes (DEGs) in the *P. pachyrhizi* genomes K8108, MT2006 and UFV02.

a DEGs were hierarchical clustered by treatment, applying hclust method using R package[63]. Differentially expressed genes in the different conditions (1) Spore; (3) appressorium *in vitro*; (4) appressorium *in planta*; (5) 10-12 HPI; (6) 24 HPI; (7) 36 HPI; (8) 72 HPI; (9) 168 HPI; (10) 192-196 HPI; (11) 288 HPI relative to the germinated spores. The scale bar shows log2 fold-change. **b** Principal component analysis of the transcriptomic data from three isolates.

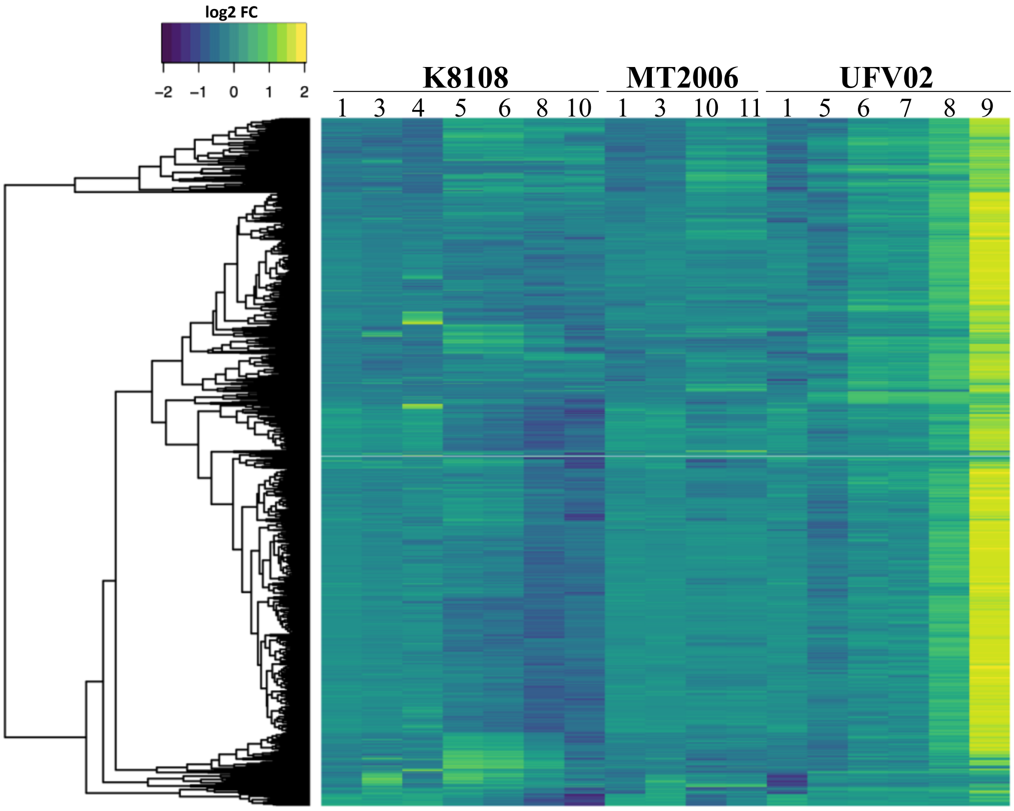

Supplementary Fig. 18. Heatmap of common DEGs between the *P. pachyrhizi* genomes K8108, MT2006 and UFV02.

DEGs were hierarchical clustered by treatment, applying hclust method using R package[63]. Differentially expressed genes in the different conditions (1) Spore; (3) appressorium *in vitro*; (4) appressorium *in planta*; (5) 10-12 HPI; (6) 24 HPI; (7) 36 HPI; (8) 72 HPI; (9) 168 HPI; (10) 192-196 HPI; (11) 288 HPI relative to the germinated spores. The scale bar shows log2 fold-change.

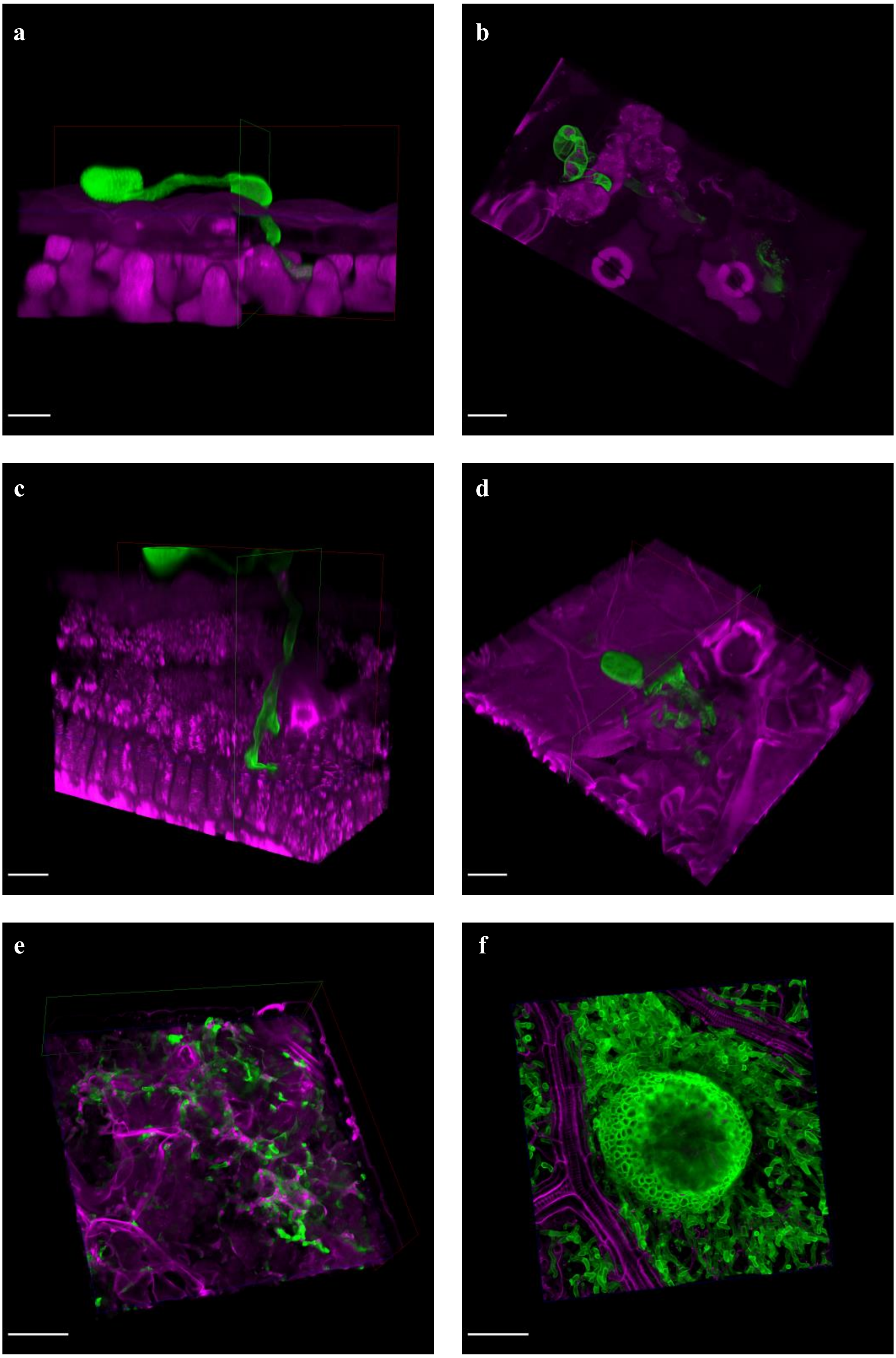
a
b
c
d
e
f

Supplementary Fig. 19. Microscopic images of soybean leaf tissue (magenta) infected with *P. pachyrhizi* (green) at different time points of the infection.
a 12 HPI, **b** 24 HPI, **c** 32 HPI, **d** 72 HPI, **e** 168 HPI, and **f** 192 HPI. Shown are 3D images obtained from z-stacks. Red, green and blue frames indicate sites of clipping to reveal areas inside the leaf. Representative micrographs are shown from three independently performed assays with similar results. Scale bars represent 20 µm (A, B, C, D) and 50 µm (E, F).

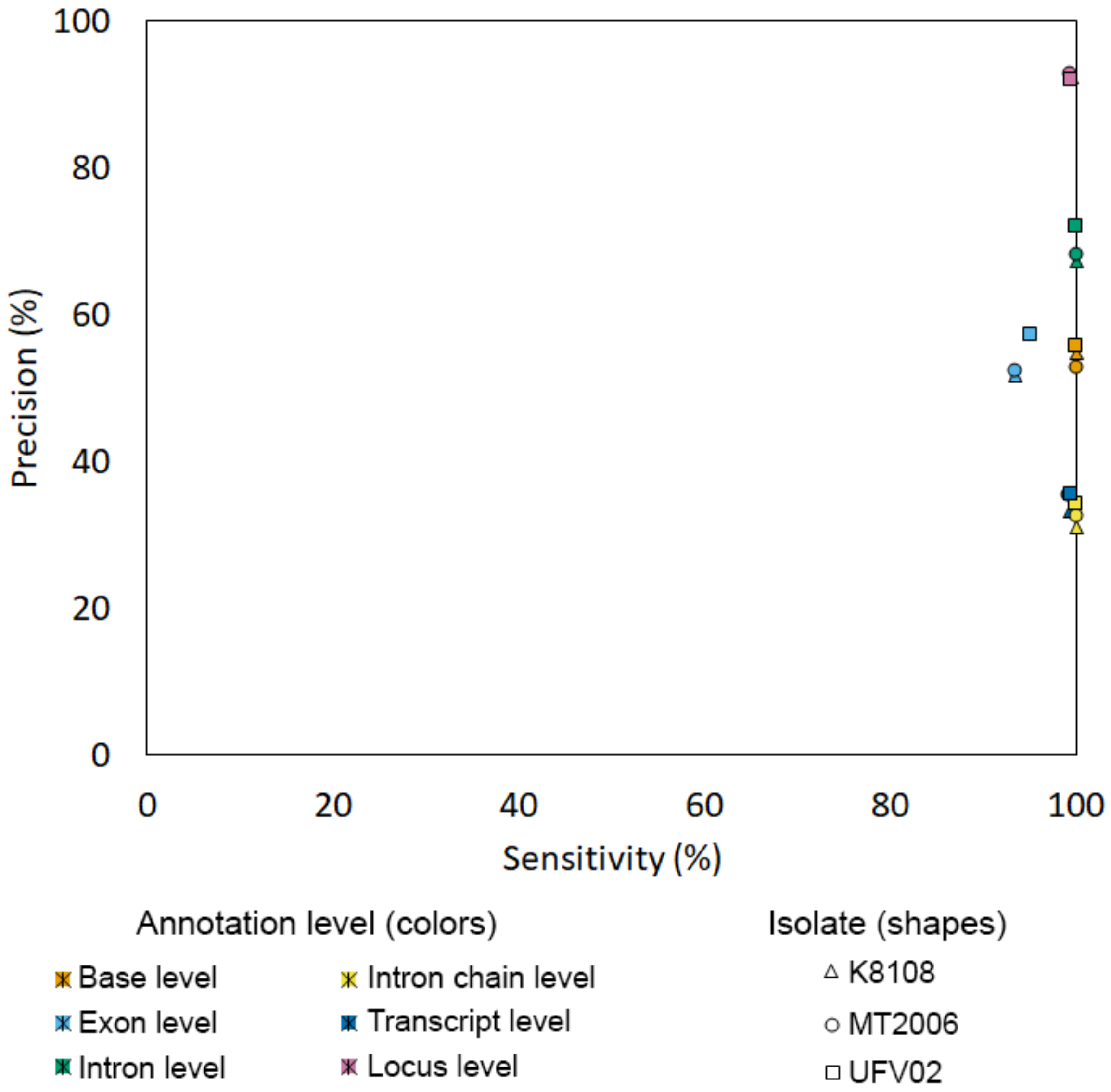

Supplementary Fig. 20. Validation of gene annotation based on expression data from the *P. pachyrhizi* genomes K8108, MT2006 and UFV02.

a

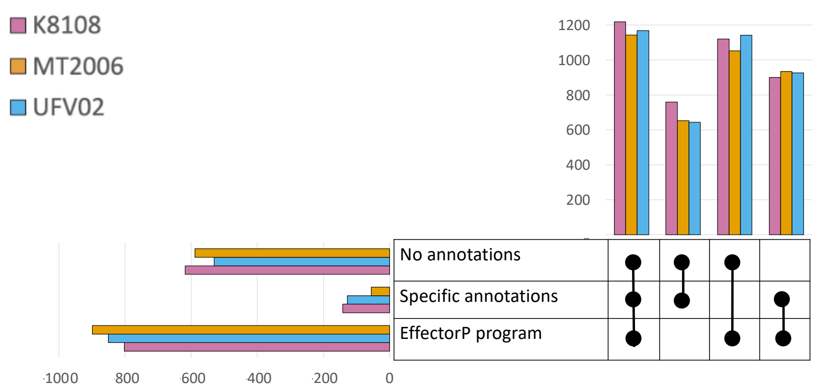

b

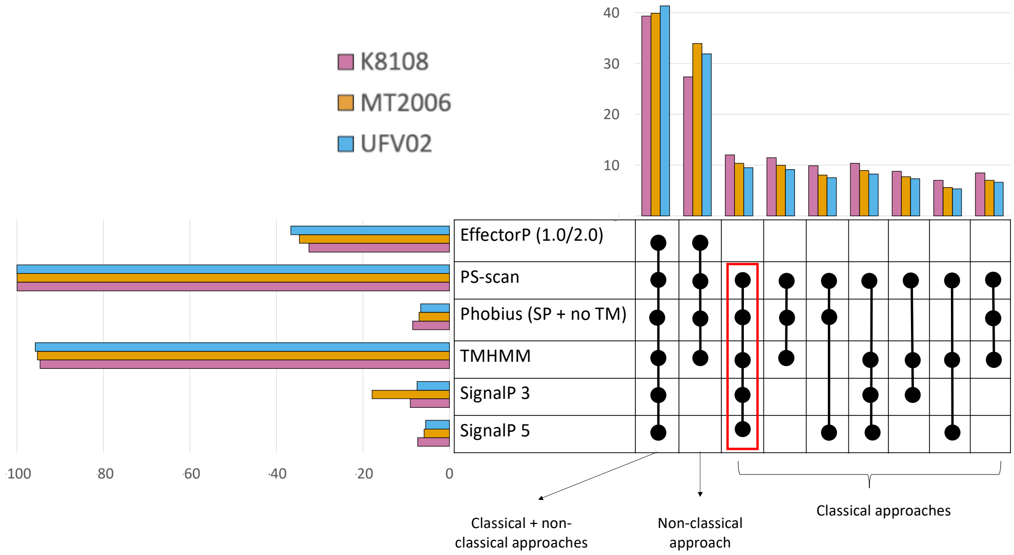

Supplementary Fig. 21. Prediction of secreted proteins from the *P. pachyrhizi* genomes K8108, MT2006 and UFV02 using different effector prediction tools.

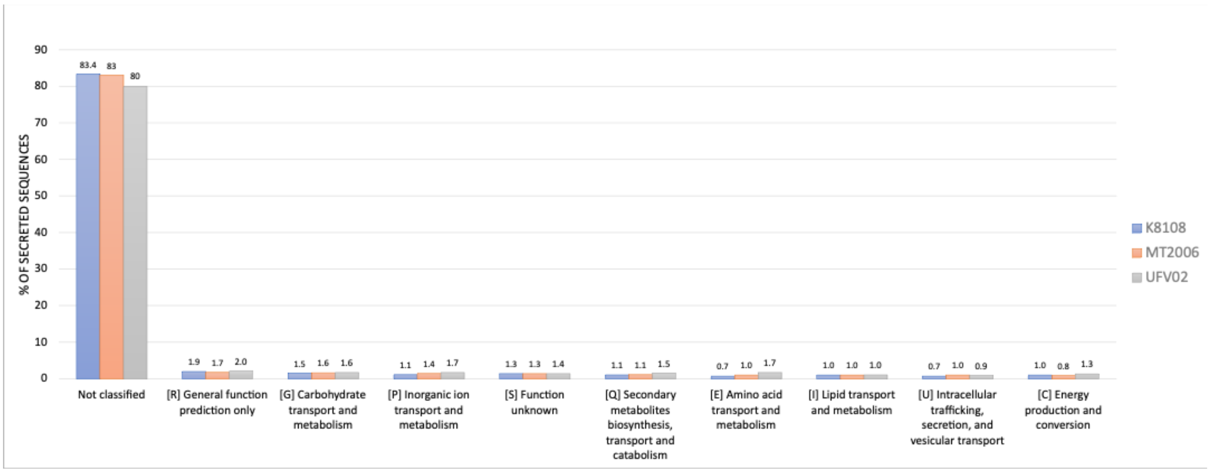

Supplementary Fig. 22. Gene categories of the secreted proteins from the *P. pachyrhizi* genomes K8108, MT2006 and UFV02.
