# Supplementary material for: Major proliferation of transposable elements shaped the genome of the soybean rust pathogen Phakopsora pachyrhizi
Source: Nat Commun. 2023 Apr 1;14:1835. doi: 10.1038/s41467-023-37551-4 (PMC10067951; doi:10.1038/s41467-023-37551-4)
Supplement: Supplementary file 3 — Description of Additional Supplementary Files [file 41467_2023_37551_MOESM3_ESM.pdf]

Description of Additional Supplementary Files

File Name: Supplementary Data 1
Description: Summary metrics of TE annotation in the *P. pachyrhizi* genomes K8108, MT2006 and UFV02.

File Name: Supplementary Data 2
Description: Complete TE annotation in the *P. pachyrhizi* genomes K8108, MT2006 and UFV02.

File Name: Supplementary Data 3
Description: Conserved TEs in the *P. pachyrhizi* genomes K8108, MT2006 and UFV02.

File Name: Supplementary Data 4
Description: Intermediate TEs in the *P. pachyrhizi* genomes K8108, MT2006 and UFV02.

File Name: Supplementary Data 5
Description: Divergent TEs in the *P. pachyrhizi* genomes K8108, MT2006 and UFV02.

File Name: Supplementary Data 6
Description: List of candidate effectors from K8108 isolate.

File Name: Supplementary Data 7
Description: List of candidate effectors from MT2006 isolate.

File Name: Supplementary Data 8
Description: List of candidate effectors from UFV02 isolate.

File Name: Supplementary Data 9
Description: Expression profile of common secreted genes in the 3 Phapa transcriptomes.

File Name: Supplementary Data 10
Description: Number of expressed TEs in the *P. pachyrhizi* genomes K8108, MT2006 and UFV02 per order and superfamily.

File Name: Supplementary Data 11
Description: Number of expressed TEs per conditions in the *P. pachyrhizi* genomes K8108, MT2006 and UFV02.

File Name: Supplementary Data 12
Description: Expression of TEs under different conditions in K8108 isolate.

File Name: Supplementary Data 13
Description: Expression of TEs under different conditions in MT2006 isolate.

File Name: Supplementary Data 14
Description: Expression of TEs under different conditions in UFV02 isolate.

File Name: Supplementary Data 15a
Description: Summary of SNPs identified using WGS Illumina data in the *P. pachyrhizi* genomes K8108, MT2006 and UFV02.

File Name: Supplementary Data 15b
Description: Summary and functional impact of variants using UFV02 as a reference genome and in the *P. pachyrhizi* genomes K8108 and MT2006.

File Name: Supplementary Data 15c
Description: Predication of the SNP impact in the *P. pachyrhizi* genome.

File Name: Supplementary Data 16
Description: Haplotype phasing of the *P. pachyrhizi* genomes K8108, MT2006 and UFV02.

File Name: Supplementary Data 17
Description: Differentially expressed genes in K8108 transcriptome.

File Name: Supplementary Data 18
Description: Differentially expressed genes in MT2006 transcriptome.

File Name: Supplementary Data 19
Description: Differentially expressed genes in UFV02 transcriptome.

File Name: Supplementary Data 20a
Description: Summary of the fungal species used for the MCL and CAFÉ analysis.

File Name: Supplementary Data 20b
Description: Dated tree with time to Most Recent Common Ancestor (tMRCA).

File Name: Supplementary Data 21
Description: Distribution of contracted gene families in 15 different fungal species.

File Name: Supplementary Data 22
Description: Distribution of expanded gene families in 15 different fungal species.

File Name: Supplementary Data 23a
Description: Summary of fungal species used for the CAZyme comparisons.

File Name: Supplementary Data 23b
Description: Summary of the CAZyme profile in fungal species.

File Name: Supplementary Data 23C
Description: Summary of the CAZyme families in fungal species.

File Name: Supplementary Data 24
Description: *Summary metrics of P. pachyrhizi genome annotations and gene models.*

File Name: Supplementary Data 25
Description: Expression profile of common genes shared by the *P. pachyrhizi* trancriptomes K8108, MT2006 and UFV02.

File Name: Supplementary Data 26
Description: Allelic Correspondence among the *P. pachyrhizi* genomes K8108, MT2006 and UFV02 gene catalogues.

File Name: Supplementary Data 27
Description: Precision and sensitivity of the gene annotations in the *P. pachyrhizi* genomes K8108, MT2006 and UFV02.
