# Supplementary material for: Major proliferation of transposable elements shaped the genome of the soybean rust pathogen Phakopsora pachyrhizi
Source: Nat Commun. 2023 Apr 1;14:1835. doi: 10.1038/s41467-023-37551-4 (PMC10067951; doi:10.1038/s41467-023-37551-4)
Supplement: Supplementary file 5 — Reporting Summary [file 41467_2023_37551_MOESM5_ESM.pdf]

nature portfolio

Corresponding author(s): Dr. HP van Esse

Last updated by author(s): Feb 4, 2023

Reporting Summary

Nature Portfolio wishes to improve the reproducibility of the work that we publish. This form provides structure for consistency and transparency in reporting. For further information on Nature Portfolio policies, see our Editorial Policies and the Editorial Policy Checklist.

Statistics

For all statistical analyses, confirm that the following items are present in the figure legend, table legend, main text, or Methods section.

n/a	Confirmed	
☐	☒	The exact sample size (*n*) for each experimental group/condition, given as a discrete number and unit of measurement
☒	☐	A statement on whether measurements were taken from distinct samples or whether the same sample was measured repeatedly
☐	☒	The statistical test(s) used AND whether they are one- or two-sided<br>*Only common tests should be described solely by name; describe more complex techniques in the Methods section.*
☒	☐	A description of all covariates tested
☒	☐	A description of any assumptions or corrections, such as tests of normality and adjustment for multiple comparisons
☐	☒	A full description of the statistical parameters including central tendency (e.g. means) or other basic estimates (e.g. regression coefficient) AND variation (e.g. standard deviation) or associated estimates of uncertainty (e.g. confidence intervals)
☐	☒	For null hypothesis testing, the test statistic (e.g. *F*, *t*, *r*) with confidence intervals, effect sizes, degrees of freedom and *P* value noted<br>*Give P values as exact values whenever suitable.*
☒	☐	For Bayesian analysis, information on the choice of priors and Markov chain Monte Carlo settings
☒	☐	For hierarchical and complex designs, identification of the appropriate level for tests and full reporting of outcomes
☒	☐	Estimates of effect sizes (e.g. Cohen's *d*, Pearson's *r*), indicating how they were calculated

Our web collection on statistics for biologists contains articles on many of the points above.

Software and code

Policy information about availability of computer code

Data collection	We used long-read sequencing technology, PacBio and Oxford nanopore, to sequence P. pachyrhizi genome. Leica SP5 confocal microscope (Leica Microsystems) was used for the confocal microscopy. The electron microscopy was performed using a PP3010 cryo-SEM preparation system (Quorum Technologies, Laughton, UK) attached to a Zeiss Gemini 300 field emission gun scanning electron microscope (Zeiss UK Ltd, Cambridge, UK).
Data analysis	We have detailed all the software and analysis parameters with the clear citation in the manuscript. The tools we used in the manuscript are:<br>Mecat (with mecat2canu adaptation)<br>SMRTTools v5.1.0.26412<br>Arrow version SMRTLINK v5<br>Albacore v2.1<br>Minimap2 (v2.14)<br>Miniasm (v0.3)<br>Racon (v1.2.0)<br>Pilon (v1.23)<br>BUSCO (v5.0)<br>REPET (V3.0)<br>KAT (v2.4.1)<br>GenomeScope (v2.0)<br>GenomeTools (v1.6.1)<br>Repbase (v20.11)

Mafft (v7.471)
PHYling
RAxML-NG (v0.9.0)
PAML (v4.8)
Pandas (v.1.3.4)
Seaborn (v0.11.2)
Python (v3.9.7)
NCBI-BLAST+ (v2.7.1)
Mummer-4.0
Assemblytics
MCScanX_h
BEDtools (v2.27.0)
Trimmomatic (v0.36, 0.39)
BWA (v0.7.17)
SAMtools (v1.9)
Picard
GATK (v3.8.1)
FastQC (v0.11.5)
Kraken2
STAR (v2.7.6a)
Picard (v2.23.2)
StringTie (v2.1.2)
TEtranscript
EdgeR v.3.1
SignalP (v3.0, 5.0),
TMHMM,
Phobius
EffectorP (v1.0, 2.0)
InterProScan
HMMsearch
ApoplastP
Localizer
TargetP
WoLFPSORT
DeepLoc

For manuscripts utilizing custom algorithms or software that are central to the research but not yet described in published literature, software must be made available to editors and reviewers. We strongly encourage code deposition in a community repository (e.g. GitHub). See the Nature Portfolio guidelines for submitting code & software for further information.

Data

Policy information about availability of data

All manuscripts must include a data availability statement. This statement should provide the following information, where applicable:

- Accession codes, unique identifiers, or web links for publicly available datasets
- A description of any restrictions on data availability
- For clinical datasets or third party data, please ensure that the statement adheres to our policy

All the sequencing data of MT2006, K8108 and UFV02 isolates has been deposited at NCBI under the accession numbers PRJNA368291, PRJEB46918, and PRJEB44222, respectively. Assemblies and annotations are also available at the DOE-JGI Mycocosm Portal (https://mycocosm.jgi.doe.gov/Phakopsora/Phakopsora.info.html). All the relevant data is available at NCBI or DOE-JGI Mycocosm Portal.

Human research participants

Policy information about studies involving human research participants and Sex and Gender in Research.

Reporting on sex and gender	Not Applicable
Population characteristics	Not Applicable
Recruitment	Not Applicable
Ethics oversight	Not Applicable

Note that full information on the approval of the study protocol must also be provided in the manuscript.

Field-specific reporting

Please select the one below that is the best fit for your research. If you are not sure, read the appropriate sections before making your selection.

- [x] Life sciences
- [ ] Behavioural & social sciences
- [ ] Ecological, evolutionary & environmental sciences

For a reference copy of the document with all sections, see nature.com/documents/nr-reporting-summary-flat.pdf

Life sciences study design

All studies must disclose on these points even when the disclosure is negative.

Sample size	For genome sequencing no sample size was calculated as we sequenced three individual mono-pustule isolates collected from different locations in Brazil. We have performed the RNA-seq analysis using the data from the individual isolates. All RNAseq data analyses was based on three independent biological replicates. For the microscopic analysis at different infection stages, the assay was performed in three independent biological replicates. No statistical method was used to determine the sample sizes.
Data exclusions	All the data is provided with the manuscript and no data was excluded.
Replication	Every RNAseq and microscopy experiments was performed with three successfully performed biological replicates.
Randomization	All experimental observations were recorded without any pre-selection of groups. A this is a descriptive study and not a hypothesis driven study randomization has not been performed .
Blinding	The investigation is not blinded.

Reporting for specific materials, systems and methods

We require information from authors about some types of materials, experimental systems and methods used in many studies. Here, indicate whether each material, system or method listed is relevant to your study. If you are not sure if a list item applies to your research, read the appropriate section before selecting a response.

Materials & experimental systems

n/a	Involved in the study	
x		Antibodies
x		Eukaryotic cell lines
x		Palaeontology and archaeology
x		Animals and other organisms
x		Clinical data
x		Dual use research of concern

Methods

n/a	Involved in the study	
x		ChIP-seq
x		Flow cytometry
x		MRI-based neuroimaging
